# Central neck lymph node metastasis in oral squamous cell carcinoma at the floor of mouth

**DOI:** 10.1186/s12885-021-07958-7

**Published:** 2021-03-05

**Authors:** Songtao Zhang, Runfang Zhang, Chao Wang, Wenbo Gong, Miaomiao Xue, Lina Liu, Yuanyuan Zhang

**Affiliations:** 1 Department of Thyroid Head and Neck Surgery, Affiliated Tumor Hospital of Zhengzhou University, Zhengzhou, Henan Province China; 2 Department of General Dentistry, The First Affiliated Hospital of Zhengzhou University, No. 1 Jianshe East Road, Zhengzhou, China

**Keywords:** Level VI metastasis, Oral squamous cell carcinoma, Head and neck squamous cell carcinoma, Central neck metastasis, Neck lymph node metastasis

## Abstract

**Background:**

Our goal was to analyze the incidence of level VI metastasis in previously untreated oral squamous cell carcinoma (SCC) patients and their clinicopathological and prognostic characteristics.

**Methods:**

Oral SCC patients with level VI metastasis were retrospectively enrolled, and their demographic and pathologic features as well as their survival data were descriptively analyzed.

**Results:**

A total of 13 cases from 1875 patients were included, all patients had SCC at the floor of mouth (SCCFOM). Eight (61.5%) patients had a pT4 tumor, and all patients had a pathological N3 neck with multiple positive lymph nodes. Adverse pathologic features were present in 100% of the patients. The size of the metastatic foci in level VI ranged from 2.6 cm to 4.5 cm with a mean value of 3.2 cm, and 5 patients showed a soft tissue deposit with no lymph node component. Recurrence occurred in all patients, and 11 patients died of uncontrolled cancer within 5 years after surgery.

**Conclusion:**

Level VI metastasis in primary oral SCCFOM is rare, and its prognosis is poor.

## Introduction

Oral squamous cell carcinoma (SCC) is the most common malignancy in the head and neck [1]. It is characterized by aggressive biological behaviors and regional metastasis, and almost half of patients with oral SCC have neck lymph node metastasis at the time of diagnosis [2, 3]. The status of the neck lymph nodes is one of the most important prognostic factors [4, 5], and the survival rate is decreased by half even if there is only one positive neck lymph node [6]. Appropriate neck management is crucial in treating oral SCC. Shah et al. [7] previously summarized their experience at the Memorial Sloan-Kettering Cancer Center in treating 1081 primary patients with SCC of the upper aerodigestive tract, in which 501 oral SCC patients received 516 radical neck dissections. The authors reported that levels I, II, and III were the most common metastatic sites, and the incidences of levels IV and V were only 20 and 4%, respectively. A subsequent series of studies confirmed this significant finding [8–15].

Level VI is an important part of the neck; it is bordered by the hyoid superiorly, the suprasternal inferiorly, and the strap muscles laterally [16]. It collects lymphatic drainage from the anterior neck, larynx, hypopharynx, thyroid, trachea, and cervical esophagus and then transfers to the lymph nodes at levels II, III and IV [17]. It is widely accepted that there is great variability in the lymphatic flow in the head and neck region [18]. Level VI is also an uncommon potential metastatic site in oral SCC, which was confirmed by Likhterov et al. [19] in two patients with recurrent oral SCC, but it is never described in primary oral SCC patients. Therefore, our goal was to analyze the incidence of level VI metastasis in previously untreated oral SCC patients and their clinicopathological and prognostic characteristics.

## Patients and methods

### Ethnic consideration

The Zhengzhou University institutional research committee approved our study, and all participants signed an informed consent agreement. All methods were performed in accordance with the relevant guidelines and regulations. All procedures performed in studies involving human participants were conducted in accordance with the ethical standards of the institutional and/or national research committee and with the 1964 Helsinki Declaration and its later amendments or comparable ethical standards.

### Patient selection

From January 2000 to September 2020, the medical records of patients with surgically treated primary oral SCC were retrospectively reviewed. Enrolled patients met the following criteria: the disease was primary and treated by surgery; detailed pathologic information of the primary tumor could be obtained; level VI metastasis was confirmed by postoperative surgical pathology; and there was no SCC arising from other sites. The disease of the enrolled patients was re-staged by the 8th AJCC classification. Information regarding the demography, pathology, operation, adjuvant treatment, and follow-up of the included patients was extracted and analyzed.

### Important variable definition

A lymph node at level VI was defined as positive on ultrasound if the smallest diameter was ≥10 mm, the ratio of the longest to smallest diameter was ≤2, and changes occurred in the internal anatomical structure of the lymph nodes. The findings suggesting node positivity on CT or MRI were as follows: an area with clear evidence of nonfat, low-density, or liquid components; a largest diameter > 15 mm at level II and > 10 mm at other levels; and a ratio of the longest to smallest diameter ≤ 2. All pathological sections were re-reviewed by at least two pathologists in a double-blind manner. Perineural invasion (PNI) was considered to be present if tumor cells were identified within the perineural space and/or nerve bundle; lymphovascular infiltration (LVI) was positive if tumor cells were noted within the lymphovascular channels [20, 21]; extranodal extension (ENE) was positive if tumor cells were noted outside the capsule. The pathologic depth of invasion (DOI) was measured from the level of the adjacent normal mucosa to the deepest point of tumor infiltration, regardless of the presence or absence of ulceration [22].

### Surgical principle

In our cancer center, systemic ultrasound, CT, MRI and/or PET-CT examinations were routinely performed for every patient. All oral SCC operations were performed under general anesthesia. The primary tumor was completely excised with at least a 1 cm margin; if necessary, a pedicled flap or free flap was used to close the defect. Neck dissection was usually performed except for tumors with very small sizes in the upper gingiva; levels of I to III/IV were manipulated for a cN0 neck, and levels of I to V were manipulated for a cN+ neck. Level VI was only dissected if metastasis was confirmed by preoperative fine or core needle aspiration biopsy. Adjuvant treatment was suggested if T3/4 disease, cervical nodal metastasis, PNI, LVI, or positive margins were present.

### Statistical analysis

Descriptive analysis was used to analyze the patients, and locoregional recurrence referred to a recurrence that occurred locally, regionally, or locally and regionally simultaneously. Distant metastasis referred to metastasis occurring in the lung or other sites. The Kaplan-Meier method (log-rank test) was used to draw the locoregional control (LRC) and disease-specific survival (DSS) curves. All statistical analyses were performed using SPSS 20.0.

## Results

A total of 13 cases from 1875 patients with surgically treated primary oral SCC were enrolled for analysis, with an incidence of 0.69%. All of the patients were male, and their mean age was 57.2 years, with a range from 43 to 67.

### Clinical data

The preoperative data of the 13 patients are presented in Table 1. All patients had SCC at the floor of mouth (SCCFOM) with midline crossing, with an incidence of 3.8% (13/342) in male patients with oral SCCFOM. Eight (61.5%) patients had a cT4 tumor, and 5 (38.5%) patients had a cT3 tumor. Clinically, 13 (100%) patients had a level I metastasis, among whom 4 patients had unilateral neck metastasis, and 9 patients had bilateral neck metastasis, and 11 (84.6%) patients had level II metastasis, among whom 8 patients had unilateral neck metastasis, and 3 patients had bilateral neck metastasis while 11 (84.6%) patients had level III metastasis, among whom 9 patients had unilateral neck metastasis and 2 patients had bilateral neck metastasis, and 6 (46.2%) patients had level IV metastasis, among whom 5 patients had unilateral neck metastasis, 1 patient had bilateral neck metastasis, and 2 patients had unilateral level V (15.4%) metastasis.

**Table 1:** Clinical data of the 13 patients with level VI metastasis

Number	Age	Sex^a^	cT	Clinical status of lymph node						Cross midline
				I	II	III	IV	V	VI	
1	67	M	4	+	+	+	+	+	+	+
2	57	M	3	+	+	+	–	–	+	+
3	43	M	3	+	–	+	+	–	+	+
4	63	M	4	+	+	+	–	–	+	+
5	62	M	4	+	+	+	+	–	+	+
6	59	M	3	+	–	+	+	–	+	+
7	61	M	4	+	+	+	–	–	+	+
8	57	M	3	+	+	+	–	–	+	+
9	48	M	4	+	+	–	+	–	+	+
10	55	M	4	+	+	+	–	+	+	+
11	65	M	3	+	+	–	+	–	+	+
12	49	M	4	+	+	+	–	–	+	+
13	58	M	4	+	+	+	–	–	+	+

^a^*M* male

### Pathologic data

All patients underwent primary tumor excision and radical or modified radical neck dissection from level I to VI. Postoperative data of the 13 patients are presented in Table 2. Eight (61.5%) patients had a pT4 tumor, and 5 (38.5%) patients had a pT3 tumor. The differentiation was well in three (23.1%) cases, moderate in five (38.5%) cases, and poor in five (38.5%) cases. Pathologically, 13 (100%) patients had level I metastasis, among whom 3 patients had unilateral neck metastasis, and 10 patients had bilateral neck metastasis, 11 (84.6%) patients had level II metastasis, among whom 9 patients had unilateral neck metastasis, and 2 patients had bilateral neck metastasis, 9 (69.2%) patients had level III metastasis, among whom 7 patients had unilateral neck metastasis, and 2 patients had bilateral neck metastasis, while 8 (61.5%) patients had level IV metastasis, among whom 7 patients had unilateral neck metastasis, 1 patient had bilateral neck metastasis, and 1 patient had unilateral level V (7.8%) metastasis. PNI and LVI were noted in 6 (46.2%) and 9 (69.2%) patients, respectively. ENE occurred in 100% of the patients. Two (15.4%) patients had a positive margin. The size of the metastatic foci in level VI ranged from 2.6 cm to 4.5 cm with a mean value of 3.2 cm, and 5 patients showed a soft tissue deposit with no lymph node component.

**Table 2:** Pathologic data of the 13 patients with level VI metastasis

Number	pT	PNI^a^	LVI^b^	Pathologic status of lymph node						ENE^c^
				I	II	III	IV	V	VI	
1	4	+	+	+	+	+	+	+	+	+
2	3	–	+	+	+	+	–	–	+	+
3	3	+	+	+	–	+	+	–	+	+
4	4	–	–	+	+	–	+	–	+	+
5	4	+	+	+	+	+	+	–	+	+
6	3	–	–	+	–	+	+	–	+	+
7	4	–	–	+	+	+	–	–	+	+
8	3	+	+	+	+	+	–	–	+	+
9	4	–	+	+	+	–	+	–	+	+
10	4	+	+	+	+	–	+	–	+	+
11	3	–	–	+	+	–	+	–	+	+
12	4	+	+	+	+	+	–	–	+	+
13	4	–	+	+	+	+	–	–	+	+

^a^ *PNI* perineural invasion, ^b^ *LVI* lymphovascular invasion, ^c^ *ENE* extranodal extension

### Possible predictor for level VI metastasis in male patients oral SCCFOM

Table 3 compared the clinical and pathologic variable differences between patients with level VI metastasis and patients without level VI metastasis, and it was noted that level VI metastasis was likely to be associated with advanced stage disease and presence of LVI and ENE.

**Table 3:** Possible predictor for level VI metastasis in male patients with oral squamous cell carcinoma at the floor of mouth

Variables	level VI metastasis		*p*
	Positive (*n* = 13)	Negative (*n* = 329)	
Age			
<40 years	0	30 (9.1%)	
≥ 40 years	13 (100%)	299 (90.9%)	0.392
cT stage			
T1 + T2	0	157 (47.7%)	
T3 + T4	13 (100%)	172 (52.3%)	0.001
cN			
N0	0	150 (45.6%)	
N1 + N2	9 (69.2%)	146 (44.4%)	
N3	4 (30.8%)	33 (10.3%)	0.002
PNI^a,d^			
Positive	6 (46.2%)	66 (20.1%)	
Negative	7 (53.8%)	198 (60.2%)	0.107
LVI^b,e^			
Positive	9 (69.2%)	50 (15.2%)	
Negative	4 (30.8%)	210 (63.8%)	< 0.001
Differentiation			
Well	3 (23.1%)	148 (45.0%)	
Moderate	5 (38.5%)	115 (35.0%)	
Poor	5 (38.5%)	66 (20.1%)	0.210
ENE^c, f^			
Positive	13 (100%)	25 (7.6%)	
Negative	0	199 (60.5%)	< 0.001

^a^ *PNI* perineural invasion, ^b^ *LVI* lymphovascular invasion, ^c^ *ENE* extranodal extension

^d^ Status of PNI in 65 (19.8%) patients remained unknown;

^e^ Status of LVI in 69 (21.0%) patients remained unknown;

^f^ Status of ENE in 105 (31.9%) patients remained unknown

### Survival data

**Fig. 1:**
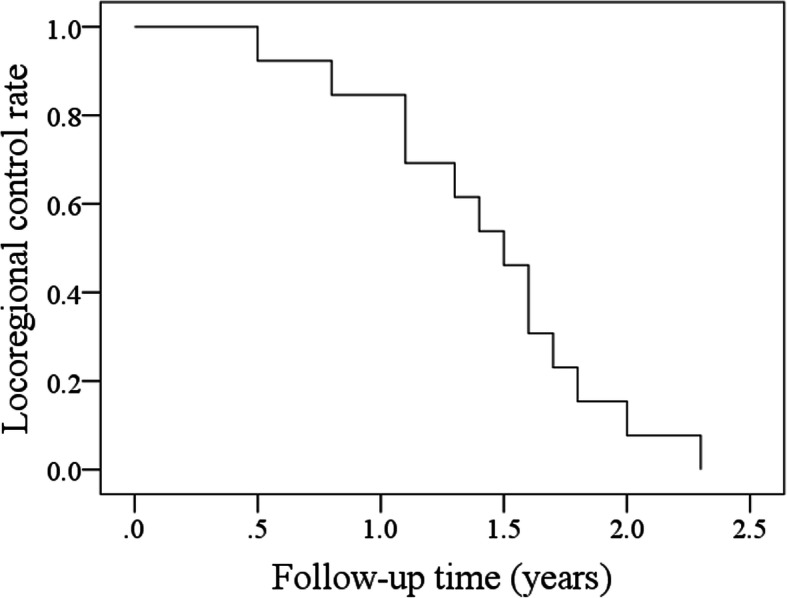
Locoregional control rate in oral squamous cell carcinoma at the floor of mouth patients with level VI metastasis

**Fig. 2:**
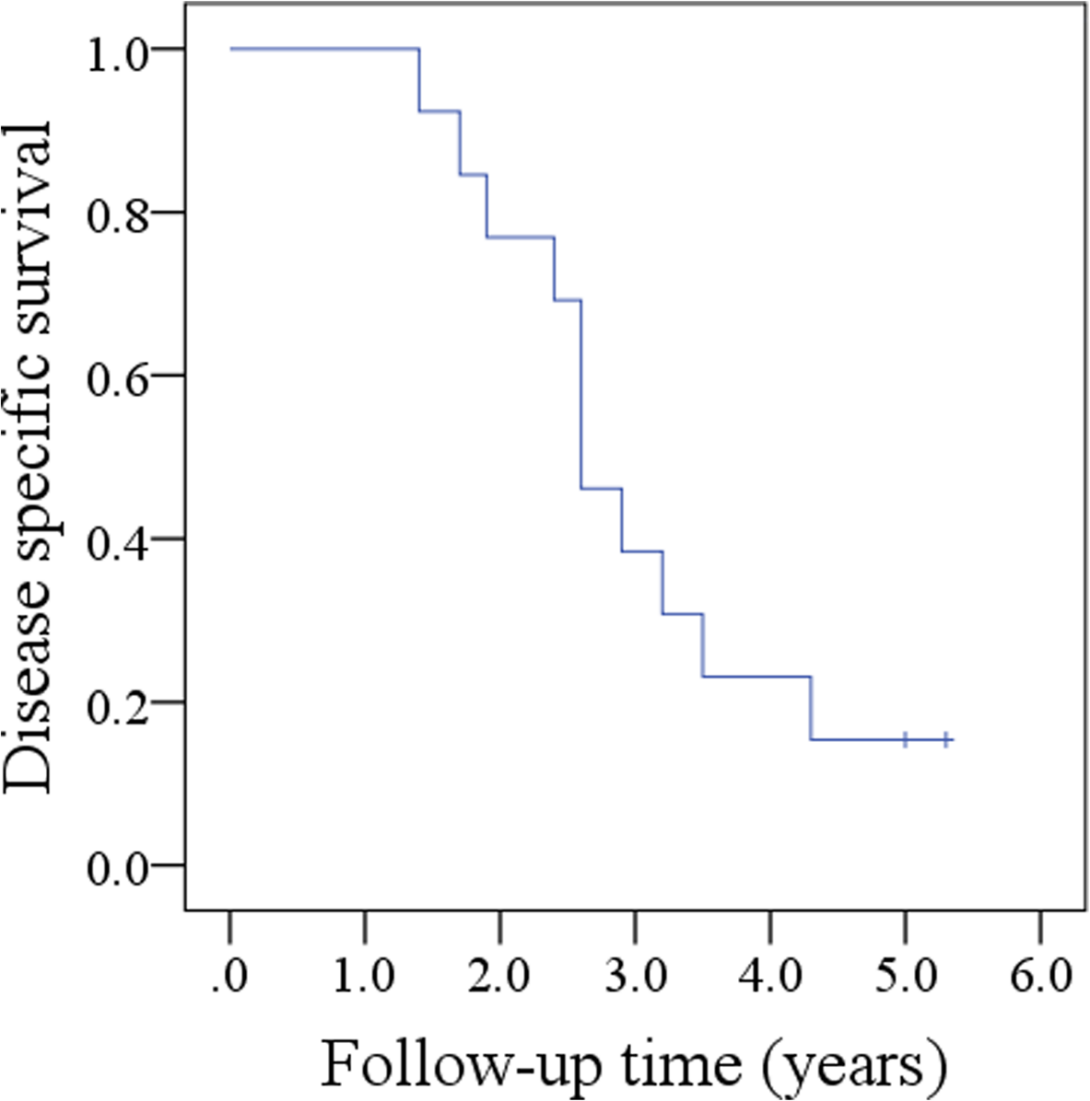
Disease specific survival in oral squamous cell carcinoma at the floor of mouth patients with level VI metastasis

All patients received adjuvant chemoradiotherapy. During our follow-up with a mean time of 2.9 (range: 1.4–5.3) years, all patients had locoregional recurrence, of whom 12 patients developed a recurrence within 2 years after surgery (Fig. 1), and concurrently, 4 patients had distant metastasis to the lungs. Two patients received salvaged surgery, and the rest received palliative chemotherapy combined with or without targeted therapy. Eleven patients died of uncontrolled cancer within 5 years after the surgery (Fig. 2), and two patients remained alive with disease.

## Discussion

The most significant finding in the current study was that the incidence of level VI metastasis in oral SCC was extremely low, and it mainly affected patients with SCCFOM. Patients with level VI metastasis usually have advanced-stage disease and multiple adverse pathological features, and their prognosis is poor.

Level VI metastasis in oral SCC is extremely rare, and Likhterov et al. [19] is the only author to previously describe this phenomenon. A female patient with T2N2b tongue SCC received primary tumor excision and modified radical neck dissection of levels I to V followed by adjuvant chemoradiotherapy; nearly 7 years later, the patient developed level VI metastasis diagnosed by fine needle aspiration. Another patient was a 76-year-old woman who presented with T3N1 gingivabuccal SCC, and the patient had level VI metastasis 7 years later after suffering several recurrences. The authors tried to analyze the possible mechanism for level VI metastasis. On the one hand, lymphatic drainage could be altered by previous surgical manipulation and radiotherapy. Estourgie et al. [23] reported that lymph node drainage in axillary and internal mammary nodes was changed in 68% of patients after excisional biopsies of breast masses. On the other hand, malignant cells were seeded in the central neck at the time of a prior tracheotomy, possibly because incision wounds are fertile ground for cancer cell growth [24].

However, level VI metastasis in primary oral SCCFOM has never been reported before, and we were the first to present this interesting finding. Its incidence was very low and it only represented 0.69% of all oral SCC patients, and 3.8% (13/342) in male patients with oral SCCFOM. The underlying explanation for this may be complicated. Tumor cells could spread by local invasion, lymphatics, blood vessels, and direct implantation. Local invasion and direct implantation were not the causes in our 13 patients because of the long distance between the primary tumor and metastatic sites and the fact that no operations were performed previously. Metastasis by lymphatics was the most likely mechanism. Three aspects must be taken into consideration when comprehending the uncommon metastatic location: the first aspect is drainage through existing crossing lymphatics, and the complexity of the lymphatic pattern in the head and neck is widely accepted [15, 16]; the second aspect is retrograde metastasis. According to the anatomic reports by Alex et al. [16] and Wang et al. [17], lymph collected at level VI is usually transferred into lymph nodes at levels II to IV, but in advanced neck disease, tumor emboli may occlude the afferent lymph collectors, causing misdirection of lymphatic drainage along other available pathways [25]. Our 13 patients all had a pathological N3 neck with multiple metastatic lymph nodes. The third aspect is the specific anatomic region with intensive midline crossing. All our patients had an advanced stage tumor with midline crossing, and it was characterized by drainage into both sides of the neck.

Hematogenous metastasis was another possible contributor. In those 13 patients, 5 patients had a soft tissue deposit at level VI without the presence of any lymph node component. Although a soft tissue deposit may reflect an affected lymph node, the possibility of distant metastasis foci cannot be ruled out. Mediastinum metastasis was uncommon but did exist. Probert et al. [26] depicted 5 of the 779 patients with head and neck cancer who developed mediastinum metastasis, of whom 4 cases were detected by autopsy. Similar findings were also reported by Ferlito et al. [27] and Takes et al. [28].

Our study showed that the prognosis of patients with level VI metastasis was poor, disease relapse frequently occurred even after systemic treatment, and cancer-related death approached immediately. This finding might be explained by the following: all patients were staged as stage IVa or IVb or higher, and advanced-stage tumors were usually associated with a poor survival [5]; all patients had multiple adverse pathologic features, each of which was associated with a worsened prognosis [2, 3, 6]; level VI metastasis in some patients may be a result of distant metastasis; and the size of the metastatic foci in level VI was relatively large. However, in the case report by Likhterov et al. [19], one of the two patients remained disease-free at a 2-year follow-up after surgery for level VI metastasis and adjuvant radiation as well as concurrent administration of taxotere and cetuximab, and the other had no follow-up data. Considering the small sample size in both studies, more research is needed to clarify this question.

Risk factors for level I to V metastasis in oral SCC have been widely analyzed [2–8], and common predictors include advanced-stage disease, poor differentiation, PNI, and LVI. Our findings also confirmed the detrimental effect of these variables on level VI metastasis; however, it must be kept in mind that routine central neck dissection is not acceptable in oral SCCFOM because of the extremely low metastasis rate.

It should be noted that the clinically positive lymph nodes at level VI were easy to detect, but we should rule out any possibilities of other malignancies. Level VI metastasis was common in thyroid cancer and SCC arising from the larynx, hypopharynx and esophagus [16, 17], and a systemic examination of at least the digestive and respiratory tracts may be a good idea. In our study, 4 patients underwent PET-CT scans, and the rest received sufficient CT/MRI scans and endoscopy. The omission of other malignancies was not allowed.

Limitations of this study must be acknowledged: our sample size was small, it was difficult for us to make a definite conclusion, a large multicenter study is needed to clarify the question, and there was inherent bias in the retrospective study.

In summary, level VI metastasis in primary oral SCCFOM is rare and it only affects male patients with advanced-stage disease. Patients with level VI metastasis usually have a poor prognosis.

## Data Availability

All data generated or analyzed during this study are included in this published article. And the primary data could be achieved from the corresponding author.
